# Matrix Metalloproteinases 7 and 10 Are Prognostic Biomarkers for Systemic Cardiovascular Risk in Individuals with Peripheral Artery Disease

**DOI:** 10.3390/biom15060853

**Published:** 2025-06-11

**Authors:** Ben Li, Farah Shaikh, Houssam Younes, Batool Abuhalimeh, Abdelrahman Zamzam, Rawand Abdin, Mohammad Qadura

**Affiliations:** 1 Department of Surgery, University of Toronto, Toronto, ON M5S 1A1, Canada; benx.li@mail.utoronto.ca; 2 Division of Vascular Surgery, St. Michael’s Hospital, Unity Health Toronto, University of Toronto, Toronto, ON M5B 1W8, Canada; 3 Institute of Medical Science, University of Toronto, Toronto, ON M5S 1A1, Canada; 4 Temerty Centre for Artificial Intelligence Research and Education in Medicine (T-CAIREM), University of Toronto, Toronto, ON M5S 1A1, Canada; 5 Heart, Vascular, & Thoracic Institute, Cleveland Clinic Abu Dhabi, Abu Dhabi P.O. Box 112412, United Arab Emirates; 6 Department of Medicine, McMaster University, Hamilton, ON L8S 4L8, Canada; 7 Li Ka Shing Knowledge Institute, St. Michael’s Hospital, Unity Health Toronto, University of Toronto, Toronto, ON M5B 1W8, Canada

**Keywords:** extracellular matrix, matrix metalloproteinase 10, matrix metalloproteinase 7, major adverse cardiovascular events, prognosis, peripheral artery disease

## Abstract

Background/Objectives: Peripheral artery disease (PAD) is associated with an increased risk of major adverse cardiovascular events (MACE), such as myocardial infarction and stroke, which are the top mortality causes in the PAD population. However, the identification of reliable biomarkers for predicting MACE in PAD patients remains limited. Proteins involved in extracellular matrix (ECM) remodeling have been implicated in atherosclerosis and may serve as potential indicators of cardiovascular risk. This study aimed to evaluate a panel of circulating proteins involved in ECM remodeling to identify those predictive of 2-year MACE in individuals with PAD. Methods: A prospective cohort of 465 PAD patients was enrolled and followed for 24 months. At baseline, plasma levels of nine ECM-related proteins were quantified. The outcome of interest was a 2-year MACE, defined as a composite of myocardial infarction, stroke, or mortality. Protein level differences between MACE vs. non-MACE patients were analyzed using Mann–Whitney U tests. Cox proportional hazards models, adjusted for baseline variables (including known cerebrovascular and coronary disease), were used to determine the independent associations between each protein and 2-year MACE. Subgroup analyses were conducted for diabetic and female patients, who are known to be at high risk for adverse events. Results: The mean age of the participants was 71 (SD 10) years, with 31.1% identifying as female and 47.2% having diabetes. Over two years, 84 patients (18.1%) experienced MACE. Among the proteins analyzed, matrix metalloproteinase-10 (MMP-10) and matrix metalloproteinase-7 (MMP-7) were significantly elevated in those who developed MACE compared to those who did not: MMP-10 (710.60 pg/mL [SD 46.09] vs. 672.40 pg/mL [SD 45.04], *p* = 0.032) and MMP-7 (5.20 pg/mL [SD 4.11] vs. 4.76 pg/mL [SD 3.86], *p* = 0.048). Both independently correlated with 2-year MACE after adjustment for all baseline factors: MMP-10 (HR 1.32, 95% CI 1.16–1.51, *p* = 0.023) and MMP-7 (HR 1.17, 95% CI 1.05–2.68, *p* = 0.026). Subgroup analyses revealed that MMP-10 was associated with MACE in diabetic patients (HR 1.18, 95% CI 1.13–1.53, *p* = 0.019), while MMP-7 was associated with MACE among females (HR 1.31, 95% CI 1.15–1.69, *p* = 0.009). Conclusions: MMP-10 and MMP-7 emerged as independent biomarkers for prognosticating 2-year MACE in PAD patients, suggesting their utility in systemic cardiovascular risk stratification. Measuring these proteins could enhance clinical decision-making by identifying high-risk individuals with PAD who may benefit from multidisciplinary vascular evaluation and intensified treatment strategies, ultimately aiming to reduce cardiovascular complications in the PAD population.

## 1. Introduction

Peripheral artery disease (PAD), characterized by atherosclerotic narrowing of the arteries in the lower limbs, affects more than 200 million individuals globally [1]. The leading cause of death in patients with PAD is major adverse cardiovascular events (MACE), including stroke and myocardial infarction (MI) [2]. This elevated risk is due in part to the strong overlap between PAD and other atherosclerotic conditions like coronary artery disease (CAD) and cerebrovascular disease (CVD) [3]. These conditions share a common pathophysiological basis rooted in systemic atherosclerosis, which is due to shared risk factors such as older age, diabetes, hypertension, dyslipidemia, and tobacco use [4]. Identifying individuals with PAD who are at heightened MACE risk is therefore critical for initiating timely, multidisciplinary care and implementing aggressive cardiovascular risk management strategies [5]. A promising avenue for improving risk stratification involves the discovery of circulating biomarkers capable of predicting MACE in PAD patients. While we have previously discovered biomarkers associated with major adverse limb events (MALE) in the PAD population [6,7], the assessment of circulating proteins that can effectively prognosticate MACE in PAD patients remains underexplored.

The extracellular matrix (ECM) is a dynamic and multifaceted network of macromolecules that surrounds cells, offering both structural integrity and biochemical cues that regulate key cellular functions such as proliferation, migration, and differentiation [8]. Its remodeling is orchestrated by various circulating proteins, notably matrix metalloproteinases (MMPs), which are proteolytic enzymes capable of degrading vital ECM components like collagen, fibronectin, elastin, laminin, and proteoglycans [8]. Because these elements are essential for maintaining vascular homeostasis, dysregulated ECM remodeling can disrupt endothelial integrity and contribute to the initiation and progression of atherosclerosis [9]. There is growing evidence that ECM turnover plays a central role in cardiovascular pathologies by promoting inflammation, vascular dysfunction, and thrombogenesis [9]. Therefore, circulating proteins that mediate ECM remodeling may serve as promising biomarkers for predicting MACE in patients with PAD [9]. Among these proteins, matrix metalloproteinases such as MMP-7 and MMP-10 have emerged as key regulators in the development of cardiovascular diseases [10,11]. Indeed, at least nine circulating ECM-related proteins have been shown to be associated with various cardiovascular conditions, including PAD, CAD, and CVD [12,13,14,15,16]. Our study focused on nine such proteins, selected based on prior research highlighting their significant associations with cardiovascular risk and disease progression [12,13,14,15,16]. Although previous investigations have linked these proteins to general cardiovascular pathology, their specific predictive value for MACE in the PAD population remains largely unexamined [12,13,14,15,16]. Our earlier work has shown that certain circulating proteins can serve as effective biomarkers for PAD diagnosis and for predicting MALE [17,18]. However, their utility in prognosticating MACE in the PAD population has not yet been fully explored [17,18]. This study aimed to evaluate the prognostic potential of a broad panel of ECM remodeling proteins to support the identification of high-risk PAD patients who could benefit from intensified medical therapies targeting systemic atherosclerosis to mitigate future cardiovascular events.

## 2. Materials and Methods

### 2.1. Ethical Approval

Ethical approval was obtained from the Unity Health Toronto Research Ethics Board on 8 February 2017 (REB #16-375). Written informed consent was obtained from all participants before enrollment, and the study adhered to the Declaration of Helsinki ethical principles [19].

### 2.2. Study Design

This investigation was designed as a prognostic study, with findings reported in alignment with the TRIPOD + AI guidelines [20].

### 2.3. Recruitment

Between January 2018 and August 2019, we prospectively enrolled consecutive individuals diagnosed with PAD at the outpatient vascular clinics of St. Michael’s Hospital. The diagnosis of PAD was confirmed based on standard criteria: a Toe-Brachial Index (TBI) below 0.7 or Ankle-Brachial Index (ABI) below 0.9, in conjunction with diminished or absent pedal pulses [21]. To ensure the inclusion of stable PAD cases, individuals presenting with elevated troponin levels, acute limb ischemia, or acute coronary syndrome within the prior three months were excluded.

### 2.4. Baseline Characteristics

Key baseline variables collected in this study included demographic and clinical characteristics such as age, sex, and the presence of established cardiovascular risk factors. Hypertension was defined as a systolic blood pressure of ≥130 mmHg, a diastolic pressure of ≥80 mmHg, or the use of antihypertensive medication [22,23]. Dyslipidemia was identified by a total cholesterol level exceeding 5.2 mmol/L, triglycerides above 1.7 mmol/L, or current treatment with lipid-lowering agents [22,23]. Diabetes was determined by a hemoglobin A1c level of ≥6.5% or the use of antidiabetic medications [22,23]. Smoking status included both current and former smokers [22,23]. Additionally, existing CAD, congestive heart failure (CHF), and previous stroke were documented [22,23]. All cardiovascular risk factor definitions were based on guidelines from the American College of Cardiology [22,23].

### 2.5. Quantification of Circulating Protein Concentrations

Peripheral blood samples were obtained from each participant, and plasma levels of nine circulating proteins were quantified using the LUMINEX multiplex assay (Bio-Techne, Minneapolis, MN, USA) [24]. The LUMINEX multiplex assay was chosen because of its demonstrated accuracy in measuring plasma protein levels in line with ELISA and Western blot using less sample and labor [25,26,27,28,29,30,31]. These proteins were selected based on their established roles in ECM and tissue remodeling pathways implicated in systemic atherosclerosis and cardiovascular pathology [12,13,14,15,16]. The panel included MMP-7, 8, and 10, HtrA serine peptidase 2 (HTRA2), Serpin A2, Serpin B3, osteoactivin, regenerating islet-derived protein 3 alpha (Reg3A), and carcinoembryonic antigen-related cell adhesion molecule 1 (CEACAM-1). By evaluating this comprehensive panel of ECM-related proteins involved in cardiovascular pathophysiology, the study aimed to identify novel prognostic biomarkers in patients with PAD. To ensure accuracy and consistency, the MagPix analyzer (Luminex Corp., Austin, TX, USA) [32] was calibrated with Fluidics Verification and Calibration Bead Kits (Luminex Corp., Austin, TX, USA) [33]. All assays were conducted on the same day to minimize inter-assay variability. Both intra- and inter-assay coefficients of variation remained below 10%, confirming assay reliability. For each target protein, a minimum of 50 beads were collected and analyzed using Luminex xPONENT software version 4.3 (Luminex Corp., Austin, TX, USA).

### 2.6. Follow-Up and Outcomes

Participants attended routine follow-up visits at one- and two-year post-baseline, with additional appointments scheduled as clinically necessary. The primary endpoint of the study was the incidence of MACE within the two-year period. MACE was defined as a composite outcome comprising stroke, MI, and mortality, with events documented through direct clinical follow-up. MI was classified according to the joint consensus definition provided by the European Society of Cardiology, American College of Cardiology, American Heart Association, and World Heart Federation [34]. Specifically, it required a rise and/or fall in cardiac troponin levels, with at least one measurement exceeding the 99th percentile upper reference limit, in combination with one or more of the following criteria: (a) symptoms of myocardial ischemia; (b) new ischemic changes on electrocardiogram; (c) formation of pathological Q waves; (d) imaging confirmation of newly compromised myocardial viability or regional wall motion consistent with ischemia; or (e) angiographic or post-mortem evidence of coronary thrombosis [34]. Stroke was defined following the criteria established by the American Heart Association and the American Stroke Association [35]. It was characterized as cell death in the brain, spinal cord, or retina resulting from ischemia [35]. This could be confirmed either by (a) pathological, imaging, or objective findings within a specific vascular territory, or (b) clinical signs of localized neurological dysfunction persisting for at least 24 h or resulting in death, with other causes excluded [35]. Mortality was assessed as all-cause death during the follow-up period.

### 2.7. Statistical Analysis

Baseline characteristics and event rates for the study cohort were summarized using means with standard deviations (SD) for continuous variables and counts with percentages for categorical variables. Comparisons of plasma protein concentrations between PAD patients who experienced MACE within 2 years and those who did not were conducted using Mann–Whitney U tests. Proteins found to be differentially expressed between these groups were further evaluated for their prognostic utility. To assess the association between these proteins and the occurrence of 2-year MACE, Cox proportional hazards models were applied, adjusting for key clinical variables including age, sex, hypertension, dyslipidemia, diabetes, smoking status (past/current), CHF, CAD, and prior stroke. Given that diabetes and female sex put PAD patients at increased risk for adverse events, we performed subgroup analyses with Cox proportional hazards analysis to assess the prognostic associations between the circulating proteins and 2-year MACE in PAD patients with diabetes and female PAD patients [36,37]. Specifically, Kavurma et al. (2023) showed that females with PAD have worse clinical outcomes than men with PAD due to complex biological, clinical, and societal factors [38]. These subgroup analyses aimed to identify biomarkers with predictive value tailored to these higher-risk populations. All statistical analyses were performed using SPSS version 23 (SPSS Inc., Chicago, IL, USA) [39], with significance defined as a two-sided *p*-value of less than 0.05.

## 3. Results

### 3.1. Study Cohort Characteristics

This study included 465 patients diagnosed with PAD. The average age was 71 years (SD 10), and 145 individuals (31.1%) were female. Cardiovascular comorbidities were common within the cohort: 84.6% had hypertension, 82.3% had dyslipidemia, and 47.2% had diabetes. In terms of smoking history, 57.9% were past smokers and 23.6% were current smokers. Additionally, 4.7% had a history of CHF, 39.0% had CAD, and 19.7% had experienced a prior stroke. All patients had clinical symptoms of intermittent claudication.

### 3.2. Cardiovascular Outcomes

Over two years of follow-up, MACE developed in 84 patients (18.1%). This included 70 cases of myocardial infarction (15.0%), 22 cases of stroke (4.7%), and 5 deaths (1.2%).

### 3.3. Plasma Protein Concentrations

Of the nine circulating proteins analyzed, two—MMP-10 and MMP-7—were found to be significantly elevated in PAD patients who experienced MACE within 2 years compared to those who did not. MMP-10 levels were 710.60 pg/mL (SD 46.09) versus 672.40 pg/mL (SD 45.04), *p* = 0.032, and MMP-7 levels were 5.20 pg/mL (SD 4.11) versus 4.76 pg/mL (SD 3.86), *p* = 0.048. No significant differences were observed for the remaining proteins between the two groups (Table 1).

### 3.4. Associations Between Circulating Proteins and Cardiovascular Outcomes

After adjusting for baseline factors—including age, sex, dyslipidemia, hypertension, smoking status, diabetes, CHF, CAD, and prior stroke—plasma levels of MMP-10 and MMP-7 remained independently correlated with 2-year MACE in PAD patients. MMP-10 showed an adjusted hazard ratio (HR) of 1.32 (95% CI: 1.16–1.51, *p* = 0.023), while MMP-7 had an adjusted HR of 1.17 (95% CI: 1.05–2.68, *p* = 0.026). No statistically significant associations were observed for the other circulating proteins (Table 2). Subgroup analyses revealed that MMP-10 was significantly correlated with 2-year MACE in PAD patients with diabetes (adjusted HR: 1.18, 95% CI: 1.13–1.53, *p* = 0.019), whereas MMP-7 was significantly associated with 2-year MACE among female PAD patients (adjusted HR: 1.31, 95% CI: 1.15–1.69, *p* = 0.009).

## 4. Discussion

### 4.1. Summary of Findings

In this study, we identified MMP-10 and MMP-7 as inflammatory proteins independently associated with 2-year MACE in patients with PAD, supporting their potential utility as prognostic biomarkers. First, among the nine plasma proteins related to ECM and tissue remodeling, MMP-10 and MMP-7 were elevated in MACE vs. non-MACE patients. Second, both MMP-10 and MMP-7 remained independently correlated with two-year MACE after controlling for all baseline variables. Third, subgroup analyses revealed that MMP-10 was predictive of 2-year MACE in PAD patients with diabetes, while MMP-7 was predictive in female PAD patients, suggesting these proteins may serve as prognostic markers for specific high-risk subgroups. Overall, our findings highlight the potential clinical relevance of MMP-10 and MMP-7 in stratifying systemic cardiovascular risk among PAD patients. Identifying individuals at increased risk may enable clinicians to tailor cardiovascular risk-reduction strategies more effectively through multidisciplinary vascular specialist referrals.

### 4.2. Comparison to Existing Literature

Liu et al. reviewed the role of MMPs in cardiovascular disease, emphasizing their importance in vascular remodeling, particularly in the progression of atherosclerotic plaques [10]. Their findings suggest that MMP activation alters plaque architecture and may directly contribute to plaque rupture [10]. Martinez-Aguilar and colleagues (2015) showed that patients with PAD presented with increased levels of MMP-10 compared with healthy controls [40]. Additionally, among PAD patients, those with chronic limb-threatening ischemia had higher serum MMP-10 levels compared to those with intermittent claudication [40]. Similarly, Matilla et al. (2020) found increased circulating MMP-10 levels in patients with aortic stenosis, where it may promote calcification via Akt phosphorylation, suggesting a broader role in cardiovascular pathology [41]. Furthermore, Giagtzidis et al. (2023) observed a significant increase in MMP-7 six months following endovascular intervention for PAD, positing that vascular manipulation and trauma from angioplasty may stimulate MMP expression [42]. However, due to the small sample size of 80 patients, the study may not have had enough power to robustly assess associations with adverse outcomes [42]. Our study extends these findings by demonstrating that both MMP-10 and MMP-7 are independently correlated with two-year MACE in PAD patients. These results underscore the potential involvement of these proteins in systemic atherosclerotic disease across multiple vascular territories. Notably, our group previously linked MMP-10 and MMP-7 with MALE in PAD patients [18]. By additionally showing their association with MACE, the current study highlights the broader relevance of these proteins in cardiovascular disease progression. Collectively, these findings underscore the need for further mechanistic studies to explore the role of MMP-10 and MMP-7 in the pathophysiology of PAD, CAD, and CVD, with the long-term aim of identifying novel therapeutic targets to improve cardiovascular outcomes in patients with systemic atherosclerosis.

### 4.3. Explanation of Findings

Several biological mechanisms may explain our findings. MMPs are a class of metal-dependent enzymes secreted by various cell types, including smooth muscle cells, endothelial cells, monocytes, macrophages, and inflammatory T cells [43]. All members of the MMP family share a conserved structural architecture, characterized by a signal peptide followed by a propeptide domain [43]. The propeptide shields the active site within the catalytic domain, which is responsible for degrading substrates such as collagen and elastin [43]. MMPs are highly potent enzymes involved in ECM degradation and remodeling, processes that are critical in numerous pathological conditions [43]. In particular, the ECM is essential to cardiovascular health, contributing to the structural integrity of the heart and blood vessels, mediating cell adhesion and communication, regulating cell survival and apoptosis, and facilitating tissue remodeling during inflammation, injury, and growth [10]. In the context of atherosclerosis, MMP activity is particularly important in weakening the fibrous cap of plaques, a process that can precipitate plaque rupture and lead to acute events such as MI, stroke, or peripheral thromboembolism [10]. MMP-10 substrates include collagens II, IV, V, and IX, as well as gelatin, laminin, casein, and fibronectin, while MMP-7 targets E-cadherin, β4-integrin, TNF-α, heparin-binding endothelial growth factor, insulin-like growth factor binding proteins, and plasminogen [10]. There is substantial evidence that MMPs are upregulated in active atherosclerotic plaques, with both animal models and human coronary specimens showing MMP localization at the advancing edges of plaques [44]. These areas often contain inflammatory cells such as macrophages and T lymphocytes and correspond to regions prone to rupture or undergoing significant remodeling [10]. Inflammatory signals activate MMPs, leading to degradation of collagen and elastin, thereby compromising plaque stability and increasing rupture risk [10]. Risk factors like smoking, diabetes, and dyslipidemia promote oxidative stress within the vessel wall, further stimulating MMP activity [10]. Hemodynamic forces, such as blood pressure fluctuations, can also enhance MMP-mediated degradation of an already weakened fibrous cap [10]. Purroy et al. (2018) demonstrated that serum MMP-10 levels were associated with coronary calcification in patients with subclinical atherosclerosis, with immunostaining revealing MMP-10 expression in regions of plaque calcification [45]. Notably, more advanced plaques released higher amounts of MMP-10 compared to non-diseased segments. These findings support the role of MMP-10 in atherosclerosis, inflammation, and plaque destabilization [45]. Similarly, Abbas et al. found that plasma MMP-7 levels were elevated in patients with carotid atherosclerosis, particularly in those with recent cerebrovascular symptoms [46]. Immunohistochemical analysis localized MMP-7 to macrophages within plaques, while in vitro studies showed that tumor necrosis factor alpha, combined with hypoxia and oxidized low-density lipoprotein, markedly upregulated MMP-7 expression [46]. Elevated MMP-7 levels were independently correlated with death in individuals with carotid stenosis [46]. Taken together, these studies provide compelling evidence for the involvement of MMP-10 and MMP-7 in cardiovascular disease pathogenesis in multiple arterial beds. Their roles in ECM degradation, plaque instability, and vascular inflammation offer a mechanistic explanation for our findings and support their potential utility as prognostic biomarkers for systemic cardiovascular risk in PAD patients.

### 4.4. Implications

Measuring plasma levels of MMP-10 and MMP-7 may provide a valuable tool for identifying individuals at elevated risk for MACE, with particular utility in primary care settings [47]. General practitioners could incorporate these biomarkers into routine assessments of PAD patients to stratify cardiovascular risk [47]. Patients identified as high risk may then be referred for comprehensive evaluation by multidisciplinary teams, including neurologists, cardiologists, and vascular medicine specialists [48]. Conversely, individuals determined to be at lower risk could remain under the care of their primary care provider, focusing on optimizing risk factor management using established therapies such as statins, acetylsalicylic acid (ASA), and evidence-based lifestyle interventions [49]. Specialists could also use these findings to individualize treatment plans based on projected two-year MACE risk. For example, adding low-dose rivaroxaban improves cardiovascular outcomes in stable PAD and CAD [50]. In high-risk individuals, further imaging—such as angiographic evaluation of coronary and cerebrovascular arteries—could help detect subclinical but hemodynamically significant lesions that may benefit from early intervention [51]. In summary, our findings can enhance care delivery for PAD patients by facilitating earlier identification of high-risk individuals, improving the referral process, and informing the intensity of treatment strategies. This targeted approach may improve cardiovascular outcomes and reduce healthcare costs, thereby supporting personalized care [52].

### 4.5. Limitations

There are several limitations to our study. First, it was performed at one center, which limits the generalizability of the findings; validation in larger, multi-center cohorts is necessary. Second, outcomes were based on two years of follow-up. Given the chronic nature of PAD, CAD, and CVD, longer follow-up is needed to better elucidate the sustained prognostic value of MMP-10 and MMP-7. Our work expands on previously developed models of cardiovascular risk assessment in PAD patients from the SMART and EUCLID trials by adding valuable MMP biomarker information [53,54]. This has important potential to improve predictive accuracy and biological relevance of current models, and further comparative studies are needed to determine the added value of MMP measurements to existing clinical prediction models [53,54]. Third, the study was not sufficiently powered to evaluate the associations between these biomarkers and the individual components of MACE. Future investigations with larger sample sizes and greater event rates are essential to clarify the predictive value of MMP-10 and MMP-7 for specific outcomes such as MI, stroke, or death. Lastly, the measurement of plasma MMP-10 and MMP-7 is currently limited to research settings. Further translational and implementation research is needed to assess the feasibility, clinical utility, and cost-effectiveness of incorporating these biomarkers into routine clinical practice for PAD patients.

## 5. Conclusions

MMP-10 and MMP-7 were identified as circulating proteins involved in ECM remodeling that correlate independently with two-year MACE in PAD patients, suggesting their potential role as biomarkers for systemic cardiovascular risk. Notably, MMP-10 was significantly associated with MACE in PAD patients with diabetes, while MMP-7 showed an association in female PAD patients, underscoring their relevance in specific high-risk subgroups. These results can enhance risk stratification for MACE in PAD, potentially enabling more targeted cardiovascular risk-reduction strategies. This is crucial because cardiovascular events such as MI and stroke remain the leading causes of mortality in patients with PAD. Moreover, our findings underscore the importance of further basic science research to uncover the mechanistic roles of MMP-10 and MMP-7 in the pathogenesis of systemic atherosclerosis. A deeper understanding of these biological pathways could contribute to the development of novel, targeted therapeutic interventions for PAD, CAD, and CVD.

## Figures and Tables

**Table 1:** Plasma protein concentrations in individuals with vs. without major adverse cardiovascular events.

	No MACE (*n* = 381)	MACE (*n* = 84)	*p*-Value
**MMP-10**	**672.40 (45.04)**	**710.60 (46.09)**	**0.032**
**MMP-7**	**4.76 (3.86)**	**5.20 (4.11)**	**0.048**
Osteoactivin	14.61 (7.95)	15.20 (8.65)	0.056
MMP-8	32.56 (40.67)	35.55 (42.15)	0.065
Reg3A	30.67 (25.35)	32.79 (26.02)	0.073
HTRA2	1.09 (1.31)	1.15 (12.50)	0.081
CEACAM-1	17.74 (11.60)	18.50 (12.43)	0.088
Serpin A12	528.39 (2872.69)	550.67 (2900.45)	0.091
Serpin B3	295.09 (780.35)	310.45 (800.35)	0.102

Protein concentrations reported as mean (standard deviation), pg/mL. Bolded rows are statistically significant (*p* < 0.05). Abbreviations: MMP (matrix metalloproteinase), HTRA2 (HtrA serine peptidase 2), Reg3A (regenerating islet-derived protein 3 alpha), CEACAM-1 (carcinoembryonic antigen-related cell adhesion molecule 1), MACE (major adverse cardiovascular event).

**Table 2:** Adjusted hazard ratios for associations between circulating proteins and 2-year major adverse cardiovascular events in individuals with peripheral artery disease.

	Adjusted Hazard Ratio	95% CI	*p*-Value
**MMP-10**	**1.32**	**(1.16–1.51)**	**0.023**
**MMP-7**	**1.17**	**(1.05–2.68)**	**0.026**
Serpin B3	0.99	(0.78–1.12)	0.341
MMP-8	1.19	(1.03–1.52)	0.514
Serpin A12	1.06	(0.76–1.33)	0.548
CEACAM-1	0.95	(0.80–1.12)	0.615
Reg3A	0.97	(0.69–1.24)	0.635
HTRA2	0.95	(0.88–1.17)	0.734
Osteoactivin	1.19	(0.93–1.33)	0.792

Bolded rows are statistically significant (*p* < 0.05). Abbreviations: MMP (matrix metalloproteinase), HTRA2 (HtrA serine peptidase 2), Reg3A (regenerating islet-derived protein 3 alpha), CEACAM-1 (carcinoembryonic antigen-related cell adhesion molecule 1), CI (confidence interval).

## Data Availability

The original contributions presented in the study are included in the article; further inquiries can be directed to the corresponding author.
